# Hospital Accommodation for Diphtheria Patients

**Published:** 1910-05-07

**Authors:** E. H. Snell

**Affiliations:** Medical Officer of Health. Coventry Public Health Dept.


### Institutional Letter-Box.

### HOSPITAL ACCOMMODATION FOR DIPHTHERIA PATIENTS.

*To the Editor of* The Hospital.

Dear Sir,—In your current issue you are good enough to insert a note concerning a report of mine to the Sanitary Committee of this Council on "The Utility of Hospital Accommodation for Diphtheria." It may not be a matter of general interest that the tenor of that report is not conveyed in the note; on that account alone I should not be writing to you, but I think that it is a matter of great concern to sanitarians occasionally to review the results obtained by the wholesale expenditure of public money on their advice. The whole trend of the argument in my report goes to show that as at present practised there is no obvious advantage to the community effected by the isolation of diphtheria cases. The review of my report by your paragraphist must have been excessively cursory in that he merely reproduces the currently, and to my mind carelessly, accepted opinion that the isolation of diphtheria patients in hospital, ignoring the contacts, is quite the right thing.

Yours faithfully,

E. H. Snell, Medical Officer of Health.

Coventry Public Health Dept., May 3, 1910.

[We referred to this report merely because of its institutional interests, and did not go into the public health quesions involved. With the large subject of contact isolation we could hardly deal in a short paragraph. In the circumstances we do not think we treated the report unfairly, if cursorily, by drawing attention to the want of hospital accommodation for diphtheria patients at Coventry.—Ed. The Hospital.]

